# Correspondence

**Published:** 1867-03

**Authors:** George Abbott

**Affiliations:** White’s Corners


### Correspondence.

To the Editor of the Buffalo Medical and Surgical Journal:

My Dear Doctor:—I much regret the evident misunderstanding which occasioned my not receiving a copy of the proof-sheets of my address in the Feb'y number, as many errors appearing therein would have been corrected. The following are some of the most important mistakes:

Page 245, 21st line, for she took two, read, she took full.

" 250, 22d and 23d lines, should be read in the same sentence.

" 257, 3d line, for invaluable, read, a valuable.

" 258, between the 10th and 11th lines, read,

The following is a favorite prescription:

℞ Liq. sub. sulph. ferri minus ʒss.
Mel. despumatum,
Cremor lacti āā, ℥ss. M.

Swab or smear the throat, or inject the nostrils with the above every two or four hours, or oftener, as the condition of the case requires.

In the opening of our address, we distinctly eschewed the design of giving an epitome of the literature, or a summary of medical opinion respecting this disease, and proposed to relate only facts and experience of our own. We first met with and commenced the treatment of this disease in 1857, before there was any American medical literature upon this subject, and groping in the dark, were obliged to meet the indications for treatment upon general principles alone. After faithfully trying solutions of nitrate of silver of divers strength, we discarded them as unsafe, and adopted the solid caustic, as indicated in the text, which, conjoined with quinia and stimulants, done us most excellent service. We soon learned to use the caustic in only those cases where there was a dense membrane lying either upon plain surfaces or over ulcerated, sloughy spots, and then simply upon the membrane—never upon the adjacent tissue. The effect would be an immediate *coagulation*, *condensation* or *hardening* of the false membrane; and we soon entertained the belief that it was that property, and but slightly, if any, its *caustic*, which rendered it beneficial. Although we had not used the article in a single case in over four years, we thought only to speak duly of a remedy which, conjoined with others, had contributed so largely in carrying us successfully through with many a hard case of this disease. The text indicated that its use was attended with many difficulties, and some danger if carelessly or unskillfully applied, and that in consequence we sought other remedies, and finally upon page 258, advanced and recommended a new and unofficinal article, liq. sub. sulph. ferri minus, devoid of those escharotic properties which irritate the "already tender and enfeebled mucous membrane," stating that it was safe in use, and had given us the best of satisfaction.

Our intention was to convey the impression that though we would have the membrane *condensed*, it should be done by *unirritative* medication, and will add, when in that condition, its removal can often be effected with comfort to the patient, or little trouble or irritation; if not, let it work off of itself.

Experience has impressed my mind with the belief that this disease is peculiarly liable to affect or derange the nervous system, and I repeat, quinine has seemed to oppose a more formidable barrier to such demonstrations, than any other article we have used. We might give additional cases in support of the reason for the "faith that is in us," but think it may be better to quote other experience and opinions, to show that we are not alone in considering quinine of utility in this disease. In the translated lectures of M. Trosseau, upon diphtheria, in speaking of general or constitutional treatment, he says: "The pharmaceutical agents which I make use of are preparations of quinia and iron. When I wish to obtain a more prompt action, I substitute for the powder of cinchona the sulphate of quinia."—[Am. Med. Monthly, 1861, page 316.] Dr. A. Jacobi of New York, of whom the same journal, remarks, "his experience in this disease is probably equal to that of any man's in America," in an article upon diphtheria, says: "Of the greatest value is that powerful febrifuge, quinia. We have never seen any bad effects, but have always found a great and rapid remission of the fever."—[Am. Med. Times, Aug. 18, 1861.] Dr. S. P. Bryan in the Louisville Medical News for October, 1860, in reporting upon the treatment of diphtheria, says: "In severe cases his main dependence is placed upon quinine." "This," he says, "appeared to control the fever and arrest the progress of the disease in all instances, except the single fatal case before referred to, in from two to five days." Also, "It appears to me that quinine exerted a curative influence not inferior to that which it exerts in ordinary malarious fevers; the pulse, which before was small, feeble, and rapid, numbering in some instances from 120 to 140 beats per minute, speedily becomes under its benign influence, fuller, stronger and slower, just as we often see in congestive fever."

But as it is unnecessary to prolong this point, I will merely add, if since that late day, such a change has come over the views of general practitioners as to make it "appear remarkable" that our experience did not coincide with theirs; is it not remarkable that it had not been previously pointed out in the columns of your valuable Journal?

If our experience had been only with the disease of the benign character seen by us within the last two or three years, we might be easily seduced into the same opinion of yourself, that "diphtheria progresses favorably without much interference." But, Doctor, if you will visit with us the Grassman family, where four out of thirteen died, North Boston, where fourteen out of fifty-six died, the Gould families where two children and two adults out of eight died, Springville where I was told in January, 1863, that seventeen out of twenty-five had died, or many other places in the south towns of Erie county, I think you would no longer believe, it was a disease which "generally progressed favorably without much interference;" at any rate, that such was not the fact with us, in the years from 1857 to 1863, the time embraced within our tables, and constituting the body of our experience.*

We did, and do still, in an "unconditional and absolute" manner recommend topical treatment as the means par excellence to conquer this disease. But, to be understood, need we ask the definition of *topical treatment*, or insinuate that it embraces not only *nitrate of silver*, but everything applied to the local demonstrations of the disease, from the mildest salt and water gargle, to the roughest action of powerful escharotics? To us, it "appears remarkable" that one of the astute perceptions of our honored friend, should have so confounded the one with the other. When Doctors disagree, who shall decide?

I have to thank you for your criticism of that, my first public production, as it has proved of much value to me.

I am, in great kindness and with high regard,

Your obedient servant,

GEORGE ABBOTT, M. D.

White's Corners, March 8, 1867.

---

GANGRENE IN DIABETES.—M. Verneuil, the eminent Parls surgeon, brought this subject a short time ago before the Surgical Society, and mentioned that he had met, in private and hospital practice, within the last six months, no less than half a dozen such cases. All the patients died.—*London Lancet.*

---

* We publish the above communication from Dr. Abbott with much pleasure, and only regret that it cannot appear in nearer connection with the original paper. Such fatality hardly shows the value of remedies, but rather otherwise. Quinine is safe and proper in all asthenic disease, but is not controlling. No objection can be urged to mild astringents and tonics, topically or otherwise, but they probably do not cure diphtheria. Diphtheria, like scarlet fever, and all other diseases of the same type, may terminate favorably under all plans of treatment, or without any interference; stimulation and support are undoubtedly proper and desirable. This disease has been known under various names for more than five hundred years, and often appeared in fearfully fatal epidemics. In this country it was known and accurately described by Dr. Douglas of Boston, in 1736, and by Dr. Bard of New York, in 1789. It was called *diphtherite* by Dr. Bretoneau in 1821. It can hardly be said that we had no American literature upon this subject in 1857, though it might not have been available to the profession very generally. This is suggested in defense of American authors.—ED.

